# Climate Mitigation through Biological Conservation: Extensive and Valuable Blue Carbon Natural Capital in Tristan da Cunha’s Giant Marine Protected Zone

**DOI:** 10.3390/biology10121339

**Published:** 2021-12-16

**Authors:** David K. A. Barnes, James B. Bell, Amelia E. Bridges, Louise Ireland, Kerry L. Howell, Stephanie M. Martin, Chester J. Sands, Alejandra Mora Soto, Terri Souster, Gareth Flint, Simon A. Morley

**Affiliations:** 1 British Antarctic Survey, NERC, Madingley Road, Cambridge CB3 0ET, UK; louela@bas.ac.uk (L.I.); cjsan@bas.ac.uk (C.J.S.); gflint@bas.ac.uk (G.F.); smor@bas.ac.uk (S.A.M.); 2 School of Geography, University of Leeds, Leeds LS2 9JT, UK; james.bell@cefas.co.uk; 3 School of Biological and Marine Sciences, University of Plymouth, Plymouth PL4 8AA, UK; amelia.bridges@plymouth.ac.uk (A.E.B.); kerry.howell@plymouth.ac.uk (K.L.H.); 4 Tristan da Cunha Conservation Department, Edinburgh TDCU 1ZZ, UK; environment.policy@tdc.uk.com; 5 School of Geography and the Environment, University of Oxford, Oxford OX1 3QY, UK; alejandra.morasoto@spc.ox.ac.uk; 6 Faculty of Biosciences, Fisheries and Economics, Norges Arktisk Universitet, veg18, 9019 Tromso, Norway; terri.souster@uit.no

**Keywords:** blue carbon, marine protected area, climate change, climate mitigation, biodiversity

## Abstract

**Simple Summary:**

Solving biodiversity loss and climate change are part of the same problem; intact natural habitats can provide powerful and efficient climate mitigation if protected. Beyond the land (forests), there is little appreciation of just how important ocean nature is to climate mitigation. Carbon captured, stored and the rate at which it is buried (sequestration) by marine organisms is called blue carbon. We measured how much blue carbon occurs around the remote islands and seamounts of the Tristan da Cunha archipelago Marine Protected Zone (MPZ). We estimated that there are 300 tonnes of carbon (tC) captured in seaweed biomass each year, a proportion of which will sink and become a part of the long-term sediment carbon store. In deeper water we found a standing stock of ~2.3 million tC in the shallowest 1000 m depths, of which equivalent to 0.8 million t of carbon dioxide has the potential to be sequestered. At current carbon prices, and were it to attract blue carbon credits, £24 million worth of blue carbon can potentially be sequestered from the standing stock of this small United Kingdom Overseas Territory. This standing stock is protected and growth could, therefore, generate an additional £3.5 million worth of sequestered carbon a year, making it an unrecognized major component of the local economy. The economic return on this example MPZ probably ranks highly amongst climate mitigation schemes. The message is that placing meaningful protection to carbon-rich natural habitats can massively help society fight climate change and biodiversity loss. Nations who provide this protection should be fairly compensated, particularly where it comes at the detriment of other economic uses of marine habitats.

**Abstract:**

Carbon-rich habitats can provide powerful climate mitigation if meaningful protection is put in place. We attempted to quantify this around the Tristan da Cunha archipelago Marine Protected Area. Its shallows (<1000 m depth) are varied and productive. The 5.4 km^2^ of kelp stores ~60 tonnes of carbon (tC) and may export ~240 tC into surrounding depths. In deep-waters we analysed seabed data collected from three research cruises, including seabed mapping, camera imagery, seabed oceanography and benthic samples from mini-Agassiz trawl. Rich biological assemblages on seamounts significantly differed to the islands and carbon storage had complex drivers. We estimate ~2.3 million tC are stored in benthic biodiversity of waters <1000 m, which includes >0.22 million tC that can be sequestered (the proportion of the carbon captured that is expected to become buried in sediment or locked away in skeletal tissue for at least 100 years). Much of this carbon is captured by cold-water coral reefs as a mixture of inorganic (largely calcium carbonate) and organic compounds. As part of its 2020 Marine Protection Strategy, these deep-water reef systems are now protected by a full bottom-trawling ban throughout Tristan da Cunha and representative no take areas on its seamounts. This small United Kingdom Overseas Territory’s reef systems represent approximately 0.8 Mt CO_2_ equivalent sequestered carbon; valued at >£24 Million GBP (at the UN shadow price of carbon). Annual productivity of this protected standing stock generates an estimated £3 million worth of sequestered carbon a year, making it an unrecognized and potentially major component of the economy of small island nations like Tristan da Cunha. Conservation of near intact habitats are expected to provide strong climate and biodiversity returns, which are exemplified by this MPA.

## 1. Introduction

The value of ecosystem services, natural capital standing stock and nature-based solutions for addressing global issues such as climate change impacts and societal quality of life, are increasing and becoming more widely and openly appreciated [1,2,3]. However, for these to be realized the fate of that carbon must be genuine sequestration (i.e., carbon that is removed from the ocean or atmosphere for at least one hundred years). Yet, conservation of biodiversity and these key societal services have lagged well behind the land [4]. The combined biological and climate mitigation value of mangrove, seagrass and salt marsh habitats have been evident for some time, but their significance is limited as they only occupy small (and drastically decreasing) strips of coast globally [5]. More recently the contribution of macroalgae, such as kelp, to both biodiversity and climate mitigation goals is also being evaluated and highlighted [6,7]. The efficiency of kelp to sequester carbon to deep-water around coasts can be globally significant [7,8]. Remote islands, with small, wave-battered coastlines, have little traditionally viewed blue carbon habitats but they can have considerable cold water coral (CWC) reefs [9,10,11]. These remote islands are further from most direct anthropogenic pressures, such as commercial fishing and terrestrial pollution. Therefore, could such biodiversity and biological carbon stores around remote islands be more resilient to climate change? The twin role in conservation and climate change mitigation of remote cold water coral dominated systems seems to have been overlooked until recently [12,13].

Cold water corals are well-established as playing a major functional role in seafloor ecosystems and are among those taxa regarded internationally as indicative of vulnerable marine ecosystems (VMEs) [14,15,16]. CWC reefs provide a number of key ecosystem services, such as creation of complex 3-D seafloor habitat, which both elevates biodiversity and provides essential fish habitat [17], as well as provisioning marine genetic resources and carbon storage to sequestration pathways. We herein define blue carbon storage as carbon held within the bodies of marine organisms over the period of months to years and sequestration as for a century or longer following Bax et al. [18]. In areas beyond national jurisdiction, the UN Food and Agriculture Organisation (FAO) recommend that VMEs, including CWC reef, be protected from significant adverse impacts, following the UN Fish Stocks Agreement, and General Assembly Resolutions 59/25, 61/105 and others; though the degree to which this occurs has been variable [19]. This recommendation is primarily based upon the role of such ecosystems in enhancing benthic secondary productivity and biodiversity, and the associated benefits it affords demersal fisheries [20], but CWC reef and other VME indicator species are also a source of other valuable ecosystem services.

Through skeletal growth, scleractinian corals (both colonial, e.g., *Desmophyllum pertusum*, and solitary e.g., *Caryophyllia* spp.) fix dissolved inorganic carbon as calcium carbonate. Complex folding skeletons may make sequestration of organic carbon (sandwiched between them) more likely by reducing surface area for microbial loop breakdown on death [21]. In an ever-carbonising world, this service has begun to take on value of its own and schemes which seek to capitalise upon this value are becoming more common. In the marine environment, these schemes have typically focussed upon, often degraded, coastal habitats such as saltmarshes or mangroves, which can be up to ten times as efficient per unit area in sequestering carbon than forests [22,23]. Arguably overlooked however have been the, sometimes vast, expanses of deep-sea ecosystems which can also act as carbon sinks [12,13,21]. Although much less efficient than coastal primary producers (e.g., mangrove stands) at converting stored to sequestered carbon, deep-water habitats are much larger, so of similar overall magnitude, and some are increasing in response to climate change (are negative feedback loops) [18,21].

The UK and other nations have set carbon neutral goals by 2050 into law and thus all potential pathways to carbon sequestration are under consideration. Of these, so called nature-based solutions (defined by the IUCN as actions which protect, manage or restore natural ecosystem services) are likely to offer amongst the most efficient and cost-effective means. Many archipelagos (e.g., overseas territories) provide potentially big carbon sinks and pathways to sequestration. Mechanisms are needed to aid avoidance of decline of old growth and safeguarding established carbon sinks as this is likely to be more cost effective than restoring, rewilding and creating sinks. Remote island communities, such as Tristan da Cunha, often have very limited means for economic diversification and so depend heavily upon the marine environment for their livelihoods. Sale of carbon credits may therefore become an important and valuable means for providing vital income to these communities, particularly those such as Tristan da Cunha, which have recently created huge marine reserves that include substantial areas of CWC reef and other carbon-sequestering species. The recent Tristan da Cunha Marine Protection Strategy included designation of a very large ‘Marine Protection Zone’ (hereafter referred to in the common acronym ‘Very Large Marine Protected Area (VLMPA)’) covers a no-take zone of over 687,000 km^2^, including around 7000 km^2^ of seamounts. This, as well as other measures that will help conserve such environments in areas that are open to fishing, notably a ban on demersal trawling and other extractive activities like seabed mining, throughout its 758,000 km^2^ of marine estate.

The Tristan da Cunha Exclusive Economic Zone (EEZ) is a large maritime zone in the temperate south Atlantic that spans the subtropical convergence. It is situated on the mid-Atlantic Ridge and includes a range of deep-sea habitats, island slopes, small seamounts, and several large guyots. Mid-oceanic islands are typified as regions of upwelling of nutrient rich water. Where this upwelling reaches the photic zone then productivity can be considerably higher than in the surrounding ocean [24]. All of these areas host CWC reef, and other VMEs, to different degrees, regulated by the habitat preferences of the different coral species present, such as *Desmophyllum pertusum* and *Solenosmilia variabilis* reefs, and Caryophyllid cup corals [11]. To date, little is known about the magnitude, variability or value of the ecosystem services provided within the Very Large Marine Protected Area (VLMPA) around Tristan da Cunha. It is clear from the literature that considerable biodiversity and carbon are apparent in both the shallows in the form of macro-algae [6,8] and of animals in deeper water [24,25,26]. Here we try to evaluate these together. In the current work we hypothesise that the area <1000 m has similar magnitudes of benthic blue carbon (per unit area) to that around nearby Ascension Island, despite being temperate and much larger in area. We compare stocks of blue carbon in deep-water with coastal kelp (macroalage) and the biota composition to other isolated tropic and polar archipelagos of the South Atlantic. Finally, we investigate drivers of variability in blue carbon and estimate the value of natural capital in terms of sequestered carbon standing stock and the provision of carbon sequestering ecosystem services in the long-term, and thus societal importance of the climate mitigation component of a VLMPA.

## 2. Materials and Methods

We undertook three research cruises to the study area, the Tristan da Cunha archipelago, in 2013, 2018 and 2019. On these we partitioned time between mapping the seabed <1000 m, measuring water column characteristics (using conductivity, temperature, and depth instruments), collecting underwater images (to investigate organism type and density) and tows of a mini-Agassiz trawl (for carbon content determination). To minimize detrimental impacts within the MPA the trawl used was just 1.25 m in width, only towed for 5 min on the seabed, and used as sparingly as possible to collect representative physical specimens. The combination of these techniques can enable estimates of carbon storage held by zoobenthos in a standardized and comparable method [25,26,27]. The main body of work was examining 987 seabed images, collected across four islands and five seamounts [11]. From these we identified each organism in each image to one of thirteen functional groups to calculate a density of each. These were multiplied by the mean value of carbon in each functional group, as determined from trawl samples. The net carbon values by site can then be scaled up to estimate carbon storage throughout the archipelago (<1000 m depth). These can be converted to sequestration estimates (e.g., ~21% of zoobenthic standing stock around Ascension Island and its seamounts was considered to store its carbon for in excess of 100 years, meeting the criterion for ‘sequestration’ [18], within the same depth zone [26]). Economic values can be attributed to carbon sequestration magnitudes using an internationally recognized value, though this value is of course subject to many and varied extrinsic factors and so regularly fluctuates.

**Figure 1:**
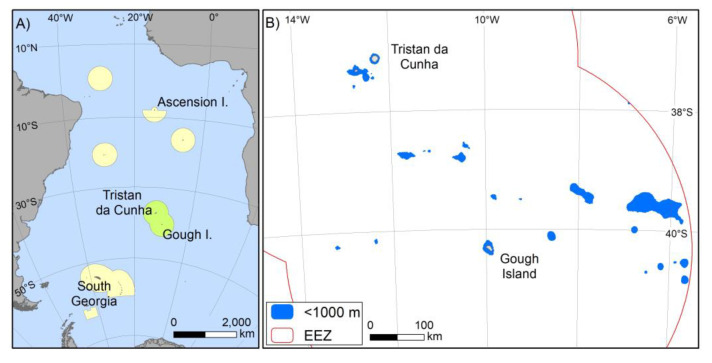
Major Marine Protected Areas in the South Atlantic Ocean (**A**) and detail of Tristan da Cunha’ s MPZ (**B**). The current study focussed on the Tristan da Cunha EEZ (shown in green in panel A), of which 91% is no take, comprising all offshore areas further than 50 nm from the northern Islands and 40 nm from Gough Island, with the exception of some areas of the larger seamounts that remain open to demersal longline fishing. Exclusive Economic Zones (red line) and locations shallower than 100 m depth (blue shade) is shown (**B**). Bathymetric data is GEBCO held by the British Oceanographic Data Centre (The GEBCO_2014 Grid, version 20150318, www.gebco.net, accessed on July 2021).

**Figure 2:**
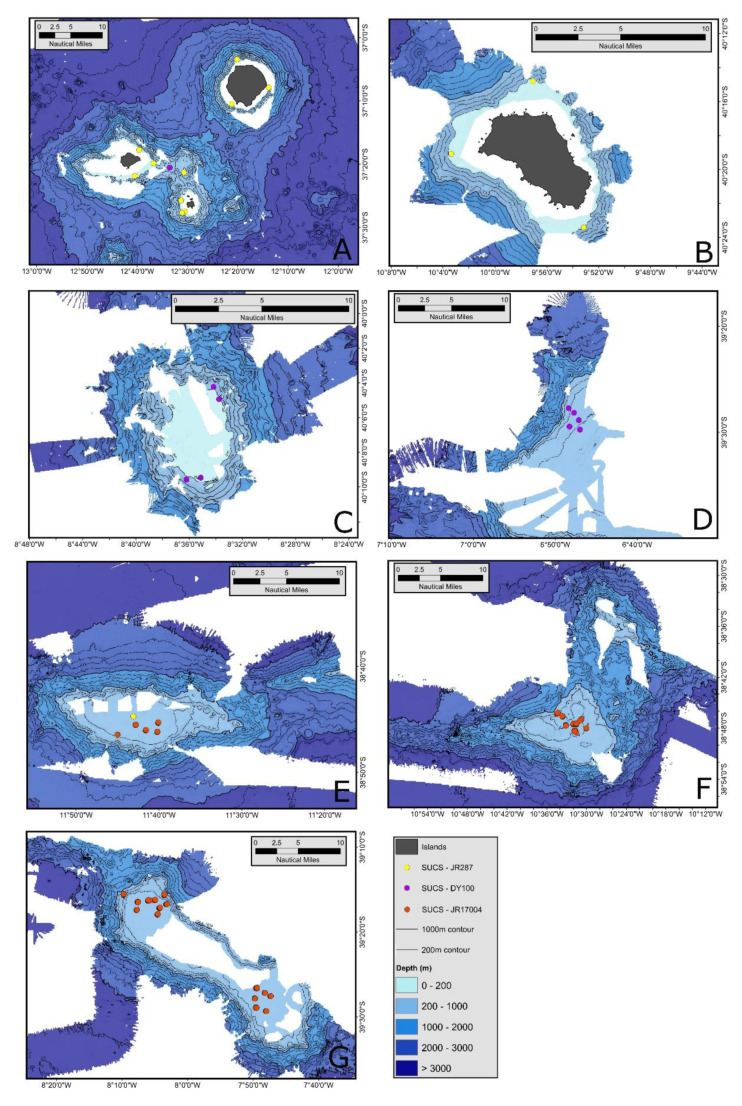
Seabed mapping and camera sites around the islands and seamounts of the Tristan da Cunha archipelago. Scale bars, depths, samples and research cruise origin of data collection are shown for each island/seamount group. The locations are Tristan da Cunha northern islands (**A**), Gough Island (**B**) and seamounts McNish (**C**), RSA (**D**), Esk (**E**), Crawford (**F**) and Yakhont (**G**).

The Tristan da Cunha archipelago is a temperate mid-Atlantic ocean series of rises, mainly comprising seamounts and guyots (Figure 1). We mapped the seabed around these localities using multibeam echosounders (EM122 and EM170, Kongsberg, Kongsberg, Noway) to identify their size and profile, and develop a sampling plan. Our spatially nested sampling protocol consisted of 11 localities (>10 km apart), with at least 3 sites at each (>1 km apart) and within these were ~20 samples (>10 m apart). The 11 localities were Tristan da Cunha, Nightingale, Inaccessible and Gough islands, and East and West Yakhont, Crawford, Esk, East and West McNish and West RSA seamounts. At each site (Figure 2) we collected replicate, haphazardly positioned seabed images (of 405.7 mm × 340.6 mm size) along a unidirectional transect using a bespoke Shelf Underwater Camera System (SUCS). This system comprises a tripod mounted camera connected by a fibre optic multimode cable to a hydraulic, ship-board winch, which in turn is connected (via a fibre optic slip-ring) to a desktop computer giving live view imagery. The 5 megapixel camera is vertically mounted, down-looking and consists of an Prosilica GC2450 body (Allied Vision, Stadtroda, Germany) equipped with a Fujinon HF12.5SA-1 lens (Fujifilm, Beford, UK). This provides ~0.1 mm measurement accuracy across the image (26 suppl). This bespoke system was specifically used because few off-the-shelf underwater camera systems are truly quantitative.

Seabed zoobenthos was measured across the entire field of view of our camera system and crucially, the camera was always positioned perpendicular to the seabed, because the tripod was landed prior to image capture. We minimized distortion by neutrality of focal length, middle F stop (F11) and a flat (rather than dome) port. Light level was reset for each image using the twin variable 2000 lumen intensity lights which were mounted on the tripod and controlled from the desktop computer. Mounting an ultra short base line (USBL) beacon on one leg of the tripod gave an accurate global positioning system (GPS) position by communication with the vessel positioning system. Matching temperature, oxygen, salinity and fluorescence data were provided by adjacent deployment of a conductivity-temperature-depth (CTD) instrument.

The identity and density of biota in images were initially processed by one observer using Image J [28] and BIIGLE 2.0 [29]. All raw images were then examined by a second observer to aid detection of cryptic biota which proved problematical to image analysis software (for example many bryozoans, hydroids and regular echinoids which cover themselves with nearby debris). All biological specimens visible in images were identified to one of 47 categories spread across 13 functional groups (recorded to at least Phylum and Class level, see Supplementary material and Table S1). This classification scheme for deep-water biota has advantages in reduced image processing time, minimising invasive, destructive sampling (to attain actual specimens of each species) and allows comparability with other deep-water, regional data sets (e.g., from Ascension seamounts, [11,26]). Assessment of biodiversity using functional groups has also been shown to have strong taxonomic surrogacy [25,27]. The thirteen functional groups were defined as follows: suspension feeder pioneers (SP), climax suspension feeders (SC), sedentary suspension feeders (SS), deposit feeding crawlers (DC), deposit feeding vermiform (DV), deposit feeding, shelled burrowers (DS), calcareous grazers (GC), scavenger/predator, sessile soft bodied (PS), scavenger/predator, sessile calcareous (PC), scavenger/predator, mobile soft bodied (PM), scavenger/predator, mobile calcareous (PL), scavenger/predator, arthropod (PA), and flexible strategy (FS) (Table 1). We used a previously tested method [25,26] to estimate the zoobenthic standing stock of organic and inorganic carbon per image. First, organism identity and density information for each image was recorded into a spreadsheet (one row per image and one column per animal identity). Corresponding oceanographic information was added from adjacent CTD casts. Substratum type (Wentworth scale) and rugosity was added by the image observer. The latter was estimated by measuring shadow length on substrata on a scale of <1 mm, 1–10 mm, 11–20 mm, 21–30 mm, 31–40 mm and 41+ mm. Densities of each functional group per image were multiplied up from the image area to 1 m^2^. These were then multiplied by the mean organic and inorganic carbon held by each functional group. These mean carbon values were calculated by drying (Agassiz trawl-collected) specimens to constant mass at 70 °C (for ~12 h), weighing, then burning at 480 °C in a furnace (~12 h) and reweighing. We assumed carbon composition constituted 50% of organic mass and 12% of skeletal (carbonate) mass [25,26]. The density of each functional group for each image was then multiplied by the mean carbon mass for the corresponding functional group so that carbon stock could be estimated for each image.

The density of each of the two main macro algae (the kelps *Macrocystis pyrifera* and *Laminaria pallida*) were taken from recent literature [30] around each of the four islands. These were multiplied by conversion factors to get from mean wet-mass to dry-mass to carbon [31,32,33]. The area of kelp canopy around each island was calculated using a kelp filter algorithm based on Sentinel 2 imagery, using an average of cloud-free images from 2015 to 2019 to cover all the possible zenith angles and tidal ranges in this lapse of time [34]. This algorithm highlights the presence of canopy reflectance on the sea surface using the difference between the red-edge (740 nm) and red (665 nm) areas of the electromagnetic spectrum. The total area per island, in hectares and square kilometres, was calculated at 20 m resolution, using the Mercator (EPSG:3857) projection. The total carbon masses of each of the two kelps were summed for each island and multiplied by the respective areas to derive a total carbon standing stock. We did not quantify export and did not account for it but such kelp may export 80% of production [35,36]. Until recently the considerable extent of macroalgal sequestration was little recognized yet now has been estimated as possibly of similar magnitude and importance to all other blue carbon habitats combined in terms of sequestration [36]. In line with literature [6,35,36,37] we estimated that the amount of carbon from macroalgae that is ultimately long term deposited in sediments (sequestration) was estimated as 11% of net primary production.

The zoobenthic composition of island and seamount sites was compared using the routine ANOSIM for analysis of similarity. The underlying structure of assemblages visualized using Bray Curtis non-metric Multidimensional Scaling (nMDS) ordination on 4th root transformed data within PRIMER (PRIMER-e Ltd., Plymouth, UK). Shade plots were used to confirm that fourth root transformation reduced the influence of highly abundant species. Because the number of pairwise comparisons was high, we used *p* < 0.01 as the threshold for significance (following Bonferroni correction factor). Following comparisons of Tristan da Cunha regional data, we then widened the comparison to include sites from around Ascension Island [26] and South Georgia [25], the data of which were collected using a similar protocol. Once standing stock of carbon was estimated for each of the 987 samples (images), we used general linear model, analysis of variance (GLM ANOVA) to investigate physical and biological factors potentially influencing carbon standing stock at site and locality levels.

Using publicly available bathymetry data, we estimated the planar area of seabed (<1000 m deep), as approximately 659.5 km^2^ around the islands and 4858.7 km^2^ of seamounts (accessible at the British Oceanographic and Polar Data Centres). This was converted to frustum surface area by multiplying by 1.5 for the seamounts and 1.9 for the island surrounds. These factors were derived by measuring the radius at 1000 m depth of a seamount and calculating the area of the cone it would make to the surface, then subtracting the area of the cone from the seamount top to the surface and finally adding the area of the seamount plateau. This resulted in a mean difference between planar and surface areas of 1.5 and 1.9 for seamounts and island surrounds respectively, to yield 1253.1 km^2^ around the islands and 7288.1 km^2^ of seamounts or a total of 8541 km^2^ seabed <1000 m deep.

To scale up zoobenthic carbon storage levels we multiplied the estimated areas of seamounts and around islands by the mean estimated zoobenthic stored carbon to give totals by area for islands and seamounts. How much of that carbon is likely to be stored long enough to become sequestered is difficult to measure or estimate (because some may not be in situ, and lifestyle and differing skeleton types of organisms alter vulnerability to microbial breakdown). Our attempted estimates of sequestration followed the protocol of previous similar work (e.g., Ascension [26], which estimated error as 1.5%). In line with UN definitions, we considered sequestration to be defined as carbon that is removed from the carbon cycle for 100+ years (thus including most of the carbon within deep-water corals [9,12]. There is a wider argument and consideration for calculation for carbon sequestration by calcareous organisms which is complex as the chemical equation to synthesise carbonate takes two molecules of carbon from water but releases one back as CO_2_. However not only is the net flux in favour of carbon build-up in these long term skeletons but having a hard skeleton increases chance of burial and reduces access for microbial breakdown of organic carbon tissues [18]. We considered benthos without hard skeletal parts as of negligible (scored as zero) sequestration potential (because of near total microbial recycling on death [26]) this included carbon in the functional groups DC, DV, PS, PM, and some SP. We multiplied carbon in the remaining functional groups by 6%, except corals and hydrocorals which were multiplied by 14.6% (of which 12% is skeletal carbon and 2.6% organic) and added carbon from dead skeleton build-ups (assuming carbon to be ~12% of carbonate by molecular mass). De Clippele et al. [38] recently calculated northern UK deep cold coral (*Desmophyllum pertusum*) to similarly comprise 5% biomass and 12% carbon ‘stock’ (=storage as termed here), in contrast to an associated sponge being 3.3% carbon stock. In contrast [38], we did not consider carbon turnover, which they calculated to be approximately 6.9% of carbon stock. Once carbon storage and sequestration levels were estimated across our 987 samples, we generated locality (seamount and island) mean values and attempted monetary valuation.

Once sequestration levels of zoobenthic carbon were estimated, monetary value can be attributed to it. Carbon markets and pricing are extremely complex and rapidly developing. The idea is to pay the ‘real cost’ of carbon, which is the costs resulting from green-house gas emissions (e.g., healthcare, change in natural disaster frequency, sea level rise etc.). There are two main approaches to carbon pricing and valuation; emissions trading systems (ETS) or carbon taxes. The ETS creates a market for total agreed emissions and therefore a value on removal of emissions by sequestration. We based valuation on the UN High level commission on carbon prices [39]. This places a value of approximately US $40+ (or £30+ in GBP) per tonne CO_2_ in 2020 but the value was envisaged to increase considerably with time to double by 2030 [39]. We multiplied the sequestered carbon estimates for the total surface area of Tristan da Cunha seabed <1000 m in depth by the UN shadow price of carbon £30 per tonne to generate the worth of standing stock natural capital. We used a regional literature value [26] of 14% for ‘ongoing ecosystem services’ through new growth and increased value of standing stock. This was based on a conservative estimate of 0.1 g·m^−2^·day^−1^ across benthic taxa, appropriate for mainly cold water coral- based systems [9,12] but very slow compared to typical reef production of approx. 2.5–7.4 g·m^−2^·day^−1^ [40].

## 3. Results

**Figure 3:**
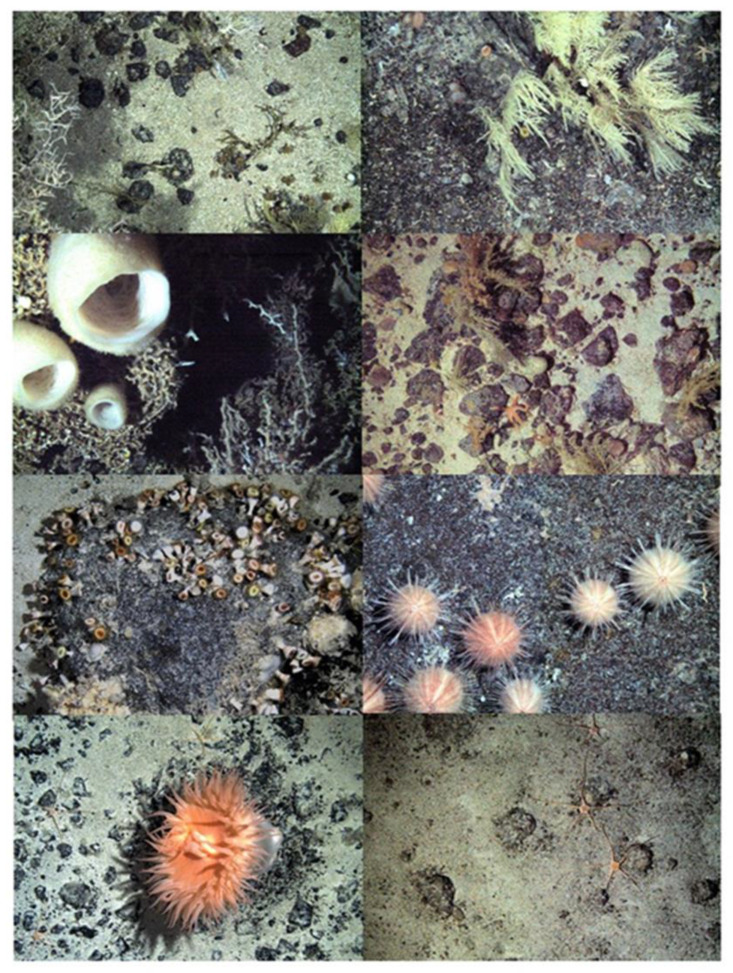
Shelf Underwater Camera System images of seabed around Tristan da Cunha’s islands and seamounts showing variability in benthic assemblages (and carbon storage).

**Figure 4:**
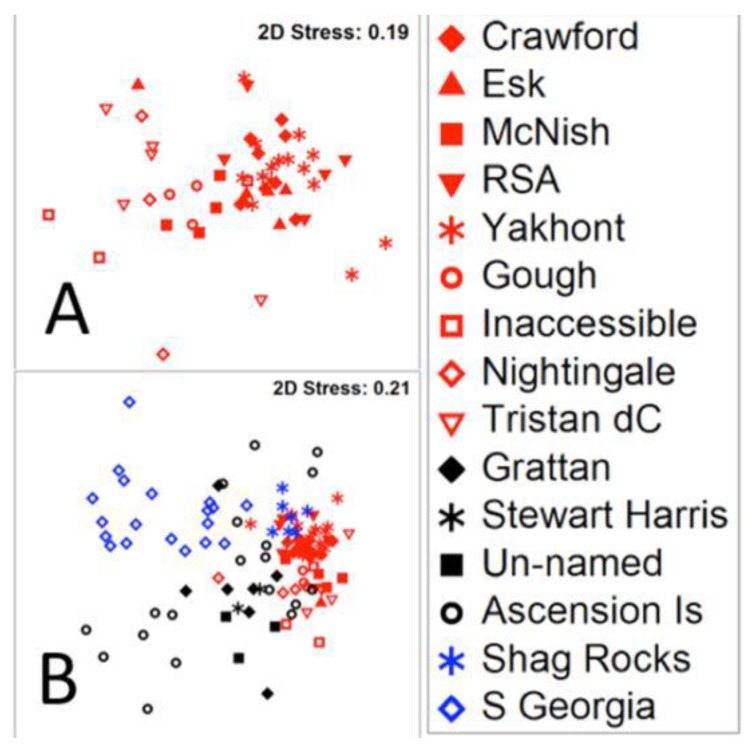
Similarity in benthic assemblage structure at <1000 m on the seabed around Tristan da Cunha (**A**) and compared with remote comparison sites (**B**). The symbols are named seamounts (solid) and islands (open), temperate (red), tropical (black) and polar (blue). Tropical and polar data [25,26].

Benthic assemblages sampled within <1000 m depth around the Tristan da Cunha EEZ and Marine Protected Area varied considerably and included 47 morphotypes across thirteen functional groups (see example assemblages in Figure 3). Four such groups (sedentary suspension feeders (SS), deposit feeding crawlers (DC), deposit feeding vermiform (DV) and scavenger/predator, mobile soft bodied (PM)) were poorly represented (Table 1). The main biological build ups around the island coasts (and Esk Guyot seamount) were cold water corals (mainly *Desmophyllum pertusum*, *Caryophyllia* cup corals, octocorals and Stylasteridae hydrocorals). In contrast, sessile soft bodied (PS, such as zooanthids, Actinarian anemone and hydroid) benthos dominated Yakhont and Crawford seamounts, and finally RSA and McNish seamounts had a more mixed benthic structure without a single group dominating. Non-metric Multidimensional Scaling (nMDS) showed the underlying dissimilarity of seamount and island assemblages (Figure 4A). ANOSIM further showed that dissimilarity patterns were complex, as Crawford was significantly distinct from islands apart from.

Inaccessible whereas Yakhont was distinct from islands apart from Gough (Table 2), of which both islands were similar distances away to both seamounts.

### 3.1. Variability of Seabed Biological Carbon Storage and Influences on It

The mean mass (g carbon) per individual (Table 3a) and per square meter (Table 3b) showed the importance of the functional groups PS, PC (anthozoan cnidarians) and to a lesser extent SC (sponges, bryozoans, and other suspension feeders). Around the islands hard corals comprised 71.4% of zoobenthic carbon standing stock, but just 16.3% at RSA seamount. Significant influences on zoobenthic carbon storage (stock) levels were seabed rugosity and substrate, biological richness (number of functional groups present), locality and site (Table 4). However, the low ANOVA F-values suggest that none of these factors explained much (of the considerable) variability, suggesting high driver complexity and inadequacy of measured factors.

We calculated that the area of seabed <1000 m within the Tristan da Cunha region is 5518 km^2^ by planar measurement and 8541 km^2^ by surface area measurement. The rugosity evident on the SUCS images and multibeam echo sounding suggests that even the latter will be a considerable underestimate of true surface area. Seamounts comprise most of this 8541 km^2^ <1000 m seabed area and dominated total, regional, zoobenthic carbon storage at 1.99 million tC (8541 km^2^ area multiplied by mean seamount carbon storage of 274 tC km^−2^) (Table 5). The equivalent value for zoobenthic carbon storage around the islands was 0.25 million tC, giving a combined total of ~2.25 million tC. We estimated that 9.3–14% of this was sequestered giving a total of ~0.22 million tC (Table 5). We consider our estimate has considerable error (−25% to +75%) because of (a) multiplying up small survey areas to large oceanic areas, (b) assuming that measured variability within and between seamounts represents the wider territory as a whole and (c) seabed area is approximately correct (but visualized rugosity suggests even surface [rather than planar] area could be a >50% underestimate).

The area of macroalgal kelp forest estimated from satellite imagery was small (5.4 km^2^ across the four islands, see Table 6). The two main kelp species (*Macrocystis pyrifera* and *Laminaria pallida*) gave an equivalent ‘snapshot’ estimate of nearly 60 °C without accounting for export (Table 6). Sequestration of this was estimated at 11% [6] to give a total of 5.5 °C.

### 3.2. Valuing Tristan da Cunha EEZ/MPA Carbon Sequestration

From the estimated total of 2.25 m t C stored in seabed organisms, we calculated that approximately 0.22 million tC will be sequestered (Table 5) and thus attract monetary value under the shadow price of carbon (~$40 or £31 GBP per tonne in 2021, see https://static1.squarespace.com/static/54ff9c5ce4b0a53decccfb4c/t/59244eed17bffc0ac256cf16/1495551740633/CarbonPricing_Final_May29.pdf accessed 16 October 2021). The CO_2_ equivalent of sequestered 0.22 million tC is 0.81 million t which approximates to a stock value of £24.4 million in year 2021 (Table 7). Ongoing ecosystem services in terms of new blue carbon generation from existing standing stock were estimated at 14% around Ascension Island, which if similar around Tristan da Cunha’s EEZ makes it worth £3.5 million each year.

## 4. Discussion

The biodiversity protected by Very Large MPA (VLMPA)s around remote mid-ocean islands has largely been to meet Convention on Biological Diversity (CBD) obligations and react to drastic global wildlife losses but also undoubtedly because of fewer objections to, and ease of, designation. Most of such protected areas are deep sea (abyssal) with few impacts mitigated by protected status, so arguably little may be altered through designation. However, remote island coasts and seamounts within these VLMPAs can be key hotspots of biodiversity, endemic species [41,42] and safeguarding banks for adjacent continental margin species [43]. From polar to tropical, they can also show moderate blue carbon ecosystem services <1000 m [26], some even with negative (mitigating) feedback loops on climate change [18]. The current study around the Tristan da Cunha archipelago shows some mid ocean VLMPAs can support considerable organismal carbon storage, amassing major banks of sequestered carbon (Table 5 and Table 7). Tristan da Cunha’s coastal carbon stock of kelp is small at an estimated 60 tC (Table 6), but surrounded by deep-water, its export (>200 tC) has higher chances of sequestration [6]. Although Tristan da Cunha’s small kelp forests are important for the islands’ economy, coastal biodiversity [30], and the most productive and efficient blue carbon sources, they are dwarfed by the magnitude of deep-water seabed communities [11]. Foley et al. [12] and Jobstvogt et al. [13] specifically highlight the potential of both biological and economic value of deep cold water coral-dominated systems, but being beyond Earth Observation (satellite) imagery this is difficult, expensive and effort intensive to do—So it has rarely been done. However, habitat suitability model outputs with suitable ground-truthing, are starting to revolutionise our ability to remotely map cold coral system distribution [11].

### 4.1. How and Why Does Mid Ocean Blue Carbon Natural Capital Vary?

The biodiversity-based designation of Tristan da Cunha’s MPA and associated conservation measures will also convey important mitigation benefits, but the blue carbon ecosystem natural capital there is complex. We found massive variability in standing stocks of seabed biological carbon around Tristan da Cunha’s islands and seamounts. For example, McNish seamount supported more than twice the blue carbon stock of nearby RSA seamount per unit area (Table 3b), which in turn was double the mean value around Ascension Island and its associated seamounts [26]. Furthermore, as around Ascension Island there could be more than two orders of magnitude difference in stocks of biological carbon within a given locality (i.e., between site). Such considerable across-scales variability may be widespread. In polar waters, similar (considerable) blue carbon standing stock variability was found between habitats around South Georgia’s continental shelf [25] and further south amongst Ross Sea seamounts [44]. The variability in blue carbon stock is reflected in the widely differing structure of regional benthic communities. For example there was considerable dispersion and overlap within <1000 m deep areas in tropical Ascension, temperate Tristan da Cunha and polar South Georgia, yet the fauna of the regions can still be seen as distinct (Figure 4B) [45,46].

Understanding the connectivity between and among habitats will be crucial to sustaining and managing blue carbon in more isolated systems as seamounts and oceanic island surrounds [7,47]. The variability between the many semi-connected islands and seamounts provides support for the considerable size of mid Atlantic MPAs, as they capture this variety in both biodiversity and diversity of ecosystem function. The factors driving such variety may be many and different between localities. Substrate type can be the major driver of benthic community and seabed carbon differences e.g., at South Georgia [25], or it’s rugosity e.g., around Ascension and its seamounts [26] or both substrate type and rugosity [48]. Elsewhere hydrography [49], such as flow rate [27] or temperature [50] can be key factors controlling the succession, growth and density of benthic communities. Focusing on cold water coral assemblages, Bridges et al. [11] found surface productivity to be the dominant factor behind deep-water benthic community differences around Tristan da Cunha, and suspension feeders certainly comprise a substantial part of the zoobenthos at all our study localities, both in terms of density (Table 1) and carbon stock (Table 3b). Likewise, carbon flux to deep fauna has been shown to be an important driver [51]. All likely contributing factors are rarely all measured at any given locality and even many that are recorded, may just be snapshots or not necessarily measured at high enough resolution and accuracy. For example, to adequately capture flow as a factor might require measuring within the boundary layer of the seabed and over a neap and spring tidal cycle—Both of which are difficult to do without in situ monitoring. It seems likely that direct anthropogenic impacts, such as past bottom trawling on some seamounts, are also likely to have had impact on the density, nature, and ecosystem service performance around Tristan da Cunha’s MPA, as found elsewhere [14,52,53,54].

### 4.2. Ongoing Blue Carbon Ecosystem Services and Sequestration

Kelp around island shorelines may be a small component of Tristan da Cunha (and other mid ocean island) blue carbon standing stock, but it contributes significantly to annual productivity and sequestration, and export (Table 6) to surrounding abyssal seabed (which comprise the vast majority of most VLMPAs). Until recently overlooked, kelp and other macroalgae are clearly important to biodiversity conservation and mitigation solutions, both regionally [8,30] and globally [6,34]. Overall, we estimated that ongoing blue carbon sequestration around Tristan da Cunha’s archipelago comprises 31 kt C year^−1^ which is the equivalent of 114 kt CO_2_ year^−1^ (0.114 million t CO_2_ year^−1^), worth approximately £3.4 million GBP annually. Seamount contributions dominate those values in our estimate, but this unsurprising as they form most of the area <1000 m deep (Figure 1B). Our estimate is likely to have very considerable error as it is based on scaling up from ‘snapshot’ observations and using (conservative) growth rate estimates rather than direct local measures (0.1 g·m^−2^·day^−1^ across benthic taxa, mean from literature [9,37,55,56]). This is twenty times less than hermatypic reef production in the Caribbean [57]. However, cold coral (e.g., *Desmophyllum pertusum*) seems quite tolerant of moderate temperature and pH change compared to shallow hermatypic corals tested on next 100-year time scale scenarios [58]. Other than kelp we did not consider near surface production but it is likely to be considerably higher than 0.1 g·m^−2^, and around Ascension waters was estimated at ~18 g carbon m^2^ [26]. Another factor which also biases us to underestimate is the lack of consideration of ecosystem services beyond 1000 m deep (but see [10]). Even if sequestration rates are two orders of magnitude less than in the upper 1000 m in the surrounding abyss, the area is more than an order of magnitude larger, so could still make an important contribution to societal goals of climate mitigation (and maintenance of biodiversity). Designation as an MPA is a strong first step to maximising mid ocean archipelago biodiversity and climate mitigation (not least by minimising the more damaging of fishing activities [14,53,59]) but ongoing effectiveness requires monitoring and management—not easy for small island economies. In theory carbon market development could help reward such practice but it will depend not just on what and how (blue) carbon is counted, but also on how strictly ‘additionality’ is considered. Additionality is whether action leads to new carbon sequestration that would have not happened otherwise. Hopefully recent literature [3,4], emphasizing that protection of near intact habitats is highest priority and most effective (than restoration or creation) in terms of carbon sequestration, should help increase valuing MPAs.

The challenge for MPA managers remains how to demonstrate that their actions demonstrably increase additionality of blue carbon capture and storage, or that extant long-term stores of carbon are inherently valuable. The value or income that is theoretically derivable from sequestration is at present conditional on the degree to which natural processes are enhanced, and nothing to do with its current status. Quite apart from the issue that this would be virtually impossible to show conclusively in deep-water habitats given available information, this creates a considerable flaw in the logic of carbon pricing. In theory, additionality (and so income) can only be gained by removing, and then restoring, part of an extant habitat, rather than preserving in its original state. Of course, some ecosystems are already degraded for many other reasons and in such case, they may be restored and so additionality increased. In the case of deep-water ecosystems however, the cost of restoring such ecosystems would likely far exceed the projected income from carbon pricing, if it is even possible. The prohibitive costs of increasing additionality is disproportionately detrimental to States whom either do not have the capacity to enhance carbon sequestration or, as in the case of Tristan da Cunha, have carbon stores that are largely in deep, offshore areas and so beyond the reach of more well-established remedial measures like mangrove reforestation. The extension of economic value from additionality (https://seea.un.org/ecosystem-accounting, accessed on 24 November 2021) to protecting existing biodiversity and the ecosystem services they already provide is now required to finance protection of some of the most intact carbon sinks.

### 4.3. What Now for VLMPAs and Mid Ocean Archipelagos?

It should be clear that protection and management of ecosystem services that still exist (such as climate change mitigation) are as, if not more, important than the creation and restoration of habitats that have been lost. Creation of many VLMPAs (such as around Tristan da Cunha) in the last two decades, and equivalents on land, has not often been followed by appropriate resources for monitoring and effective safeguarding, despite reasonable baseline survey ‘start points’ (e.g., Ascension [59] and Tristan da Cunha [11]). The current study shows the VLMPA is a major resource of considerable ‘economic’ value (if measured under shadow price of carbon), so a logical next step is making sure it is safeguarded beyond mere declaration of intent. Best practise should include light touch monitoring, making use of Earth Observation imagery but inevitably will require some frequency of ship-based non-invasive measurements.

## 5. Conclusions

We have shown that mid-ocean temperate seamounts and islands can support very considerable biological carbon stocks as well as biodiversity. Crucial next steps are measurement of how much storage is converted to sequestration per unit time (which in the case of the macro algal component would not be in situ but exported). In terms of sequestration, we have merely posited an unsupported estimate, along the lines of literature values. We have suggested what this could be worth in monetary terms and towards decarbonisation targets. We think that the protection around the above 1000 m rises in the Tristan da Cunha archipelago is an example of well targeted, meaningful, marine protection from stressors. These can strongly help address both nature loss and climate change. We must use the current urgency around climate change and nature loss to make more and better designation and implementation of protection to carbon- and species-rich environments.

## Figures and Tables

**Table 1:** Density of biodiversity by functional group and locality <1000 m depth. The data are: density in individuals or colonies/m^2^. The localities are islands of the Tristan da Cunha archipelago (Islands) and named seamounts. The functional groups are sessile suspension feeder pioneers (SP), sessile climax suspension feeders (SC), sedentary suspension feeders (SS), deposit feeding crawlers (DC), deposit feeding vermiform (DV), deposit feeding, shelled burrowers (DS), calcareous grazers (GC), scavenger/predator, sessile soft bodied (PS), scavenger/predator, sessile calcareous (PC), scavenger/predator, mobile soft bodied (PM), scavenger/predator, mobile calcareous (PL), scavenger/predator, arthropod (PA), and flexible strategy (FS). The most common functional group at each locality is shown in bold. Values of 0.1 or less are indicated by *.

Locality	SP	SC	SS	DC	DV	DS	GC	PS	PC	PM	PL	PA	FS
Islands	13	16.3	0.3	*	*	0.8	2.2	17.4	**70**	*	2.2	1.5	1.1
Yakhont	4.6	45.1	0.2	*	*	4.7	9.7	**83.9**	21.6	0.6	1.1	1.1	18.7
Crawford	18.7	43.5	*	*	*	4.4	15.5	**81**	17.5	0.3	1.4	6.2	10.2
Esk	11.9	32.5	0.2	*	*	5	8.7	31	**68.6**	1.2	0.9	3.5	6.1
McNish	9.5	36.8	*	*	*	36.3	2.7	25	**43.1**	0.9	4	1.8	1.1
RSA	6.4	**32.3**	1	*	*	15.3	5	16.9	14.6	*	1.1	0.4	15.1

**Table 2:** One way ANOSIM on assemblage differences between localities in the Tristan da Cunha region. Pairwise values shown are ANOSIM R, in which significance is <0.01 (*) and <0.001 (**). R close to zero means little pairwise difference whereas R nearing 1 is highly different. Non significant pairwise comparisons are shown with an ‘x’.

Locality	Crawford	McNish	RSA	Esk	Gough	Tristan dC	Nightingale	Inaccessible
Yakhont	x	x	x	x	x	0.72 **	0.83 **	0.68 *
Crawford		0.63 *	0.35 *	x	0.72 *	0.78 **	0.88 *	x
McNish			x	x	x	0.55 *	x	x
RSA				x	x	x	x	x
Esk					x	x	x	x
Gough						x	x	x
Tristan dC							x	x
Nightingale								x
Inaccessible								

**Table 3:** Zoobenthic carbon storage (stock) by functional group and locality <1000 m depth. The data are: mean mass g carbon; (a) per individual and zoobenthic carbon g/m^2^ = t/km^2^, (b) by functional group and locality. The Tristan da Cunha archipelago localities are as in Table 1. Values of 0.1 or less are indicated by *. The dominant functional group (by mass) for each area is shown in bold.

Locality	SP	SC	SS	DC	DV	DS	GC	PS	PC	PM	PL	PA	FS
(a) islands	0.83	1.45	1.1	0.95	0.85	1.53	2.8	1.51	2.86	0.91	3.79	1.27	0.8
seamount	0.88	1.41	1.08	0.93	0.87	2.07	2.8	1.38	2.94	0.63	3.36	1.6	0.8
overall	0.86	1.43	1.09	0.95	0.85	1.83	2.8	1.43	2.88	0.78	3.58	1.44	0.8
(b) Islands	11	23	0.3	*	*	1.4	6.8	23	**191**	*	8	2.2	0.8
Yakhont	4.1	65	0.2	*	*	8.7	38	**120**	59	0.5	3.7	1.6	15
Craw	17	65	*	*	*	7.9	23	**119**	53	0.2	5.8	9	8.3
Esk	8.9	46	0.2	*	*	9.8	26	40	**203**	1.5	2	5.2	4
McNish	9	54	*	*	*	86	3.9	35	**134**	*	16	2.3	0.2
RSA	5.1	**55**	1.1	*	*	16	16	25	26	*	2.5	0.8	12

**Table 4:** GLM ANOVA showing complex influences on zoobenthic carbon storage (stock) around the Tristan da Cunha region. Seabed roughness (rugosity), substrate, richness (number of functional groups) and spatial factors (locality and site) are significant but only explain a moderate part of data variability.

Source	DF	Adj SS	Adj MS	F	*p*
Rugosity	6	3,117,526	519,588	7.1	0.001
Substrate	8	3,773,735	471,717	6.4	0.001
Richness	9	3,993,682	443,742	6.1	0.001
Locality	9	2,886,060	320,673	4.4	0.001
Site	14	3,766,570	269,041	3.7	0.001
Temperature	1	235,935	235,935	3.2	0.073
Oxygen	1	230,455	230,455	3.2	0.076
Chlorophyll	1	22,701	22,701	0.3	0.578
Salinity	1	127	127	0.0	0.967
Error	936	68,533,741	73,220		
Total	986	103,092,216			

**Table 5:** Seabed area <1000 m depth and zoobenthic carbon around the Tristan da Cunha archipelago. Planar area is the 2-D estimate and so an underestimate of true surface area, which takes account of topography. The columns are from left to right; localities, area, zoobenthic stored carbon in tonnes per km^2^ (ZbC t/km^2^), total zoobenthic stored carbon in million tonnes (ZbC Mt), zoobenthic carbon expected to be sequestered in tonnes per km^2^ (Seq t/km^2^), total zoobenthic carbon expected to be sequestered in million tonnes (Seq million t).

Area Measure	Area km^2^	Zb tC/km^2^	Zb Million tC	Seq tC/km^2^	Seq Million tC
**Planar Area**					
Islands	659.5	200.6	0.13	28	0.019
seamounts	4859	274	1.33	25	0.124
total	5518		1.46		0.143
**Surface Area**					
Islands	1253	200.6	0.25	28	0.035
seamounts	7288	274	1.99	25	0.182
total	8541		2.25		0.217

**Table 6:** Area, density and carbon standing stock of kelp around the shallows of the Tristan da Cunha islands archipelago. The densities of kelp; *Macrocystis pyrifera* (M) and *Laminaria pallida* (L) [30]. Carbon storage of (C store) was calculated for each island as area × density × carbon mass for each of the two kelp types. This was *Macrocystis* [mean wet mass 1 kg × drymass conversion (0.115) = 0.115 kg drymass; × 0.3 carbon conversion = 0.035 kg carbon [33] + *Laminaria* (mean wet mass 0.8 kg × 0.13 = 0.104 kg dry mass, and × 0.252 = 0.026 kg Carbon [32]. Carbon export was not accounted for but could involve multiplying ‘C store’ by five [35]. Sequestration was estimated as 11% of net primary production [6].

Locality	Area km^2^	Density M × 1000	Density L × 1000	C Store Tonnes	Seq °C
Gough	2.89	54	237	23.27	2.6
Tristan dC	0.95	51	543	15.11	1.6
Inaccessible	0.69	54	387	8.25	0.5
Nightingale	0.87	44	518	13.06	0.8
Total	5.4				5.5

**Table 7:** Sequestered zoobenthic carbon, CO_2_ equivalent and its value on seabed area <1000 m depth around the Tristan da Cunha archipelago compared with that around Ascension Island. The columns are from left to right; localities, total sequestered zoobenthic carbon in million tonnes on the basis of surface area (Seq million t), their equivalent CO_2_ amount in million tonnes (CO_2_ equiv), minimum value on the shadow price of carbon in £ millions GBP (£ million), annual increment, ie ongoing ecosystem services in million tonnes carbon per year (oES tC/year), annual increment in million tonnes CO_2_ equivalent (oES milliont CO_2_), and annual increment in value, £ millions (oES).

	Seq Million tC	CO_2_ Equiv	£ Million	oES Million tC/year	oES Million tCO_2_	oES £ Million
TdC islands	0.04	0.13	3.9	0.005	0.018	0.54
seamounts	0.18	0.68	20.5	0.026	0.095	2.87
total	0.22	0.80	24.4	0.031	0.114	3.42
Ascension total	0.01	0.03	1	<0.001	0.002	0.006

## Data Availability

All data supporting reported results can be found in online supplement.

## Supplementary Materials

The following are available online at https://www.mdpi.com/article/10.3390/biology10121339/s1, Table S1: biodiversity and carbon storage of Tristan da Cunha benthos.

## References

[1] Constanza R., d’Arge R., de Groot R., Farber S., Grasso M., Hannon B., Limburg K., Naeem S., O’Neill R., Paruelo J. (1997). The value of the world’s ecosystem services and natural capital. Nature.

[2] De Groot R., Brander R., van der Ploeg S., Costanza R., Bernard F., Braat L., Christie M., Crossman N., Ghermandi A., Hein L. (2012). Global estimates of the value of ecosystems and their services in monetary units. Ecosyst. Serv..

[3] Pörtner H.O., Scholes R.J., Agard J., Archer E., Arneth A., Bai X., Barnes D., Burrows M., Chan L., Cheung W.L. (2021). IPBES-IPCC Co-Sponsored Workshop Report on Biodiversity and Climate Change.

[4] Sala E., Mayorga J., Bradley D., Cabral R.B., Atwood T.B., Auber A., Cheung W., Costello C., Ferretti F., Friedlander A.M. (2021). Protecting the global ocean for biodiversity, food and climate. Nature.

[5] Goldberg L., Lagomasino D., Thomas N., Fatoyinbo T. (2020). Global declines in human-driven mangrove loss. Glob. Chang. Biol..

[6] Krause-Jensen D., Duarte C.M. (2016). Substantial role of macroalgae in marine carbon sequestration. Nature Geoscience.

[7] Queirós A.M., Stephens N., Widdicombe S., Tait K., McCoy S.J., Ingels J., Rühl S., Airs R., Beesley A., Carnovale G. (2019). Connected macroalgal-sediment Syst.: Blue carbon and food webs in the deep coastal ocean. Ecol. Monogr..

[8] Filbee-Dexter K., Wernberg T. (2020). Substantial blue carbon in overlooked Australian kelp forests. Sci. Rep..

[9] Roberts J.M., Wheeler A.J., Freiwald A., Cairns S.J. (2010). Cold-Water Corals. The Biology and Geology of Deep-Sea Coral Habitats.

[10] La Bianca G., Tillin H., Hodgson B., Erni-Cassola G., Howell K., Rees S. (2018). Ascension Island Natural Capital Assessment: Marine Ecosystem Services Report.

[11] Bridges A.E., Barnes D.K.A., Bell J.B., Ross R.E., Howell K.L. (2021). Benthic community composition of South Atlantic seamounts. Front. Mar. Sci..

[12] Foley N., van Rensburg T., Armstrong C. (2010). The ecological and economic value of cold-water coral ecosystems. Ocean Coast. Manag..

[13] Jobstvogt N., Townsend M., Witte U., Hanley N. (2014). How Can we identify and communicate the ecological value of deep-sea ecosystem services?. PLoS ONE.

[14] Rogers A., Baco A., Griffiths H., Hart T., Hall-Spencer J., Pitcher T.J., Morato T., Hart P.J.B., Clark M.R., Haggan N., Santos R.S. (2007). Corals on seamounts. Seamounts: Ecology, Fisheries & Conservation.

[15] Kenchington E., Murillo F.J., Lirette C., Sacau M., Koen-Alonso M., Kenny A., Ollerhead N., Wareham V., Beazley L. (2014). Kernel density surface modelling as a means to identify significant concentrations of vulnerable marine ecosystem indicators. PLoS ONE.

[16] Kenny A.J., Cambell N., Koen-Alonso M., Pepin P., Diz D. (2018). Delivering sustainable fisheries through adoption of a risk-based framework as part of an ecosystem approach to fisheries management. Mar. Policy.

[17] Kaiser M.J. (2004). Fish in deep-water coral habitats. Science.

[18] Bax N., Sands C., Gogarty B., Downey R.V., Moreau C.V.E., Moreno B., Held C., Lund Paulsen M., McGee J., Haward M. (2021). Perspective: Increasing blue carbon around Antarctica is an ecosystem service of considerable societal and economic value worth protecting. Glob. Chang. Biol..

[19] Bell J.B., Guijarro-Garcia E., Kenny A. (2019). Demersal fishing in areas beyond national jurisdiction: A comparative analysis of regional fisheries management organizations. Front. Mar. Sci..

[20] Pham C.K., Vandeperre F., Menezes G., Porterio F., Isidro E., Morato T. (2015). The importance of deep-sea vulnerable marine ecosystems for demersal fish in the Azores. Deep Sea Res. Part I.

[21] McLeod G.L., Chmura S., Bouillon R., Salm M., Bjork C.M., Duarte C.E., Lovelock W., Schlesinger W.H., Silliman B.R. (2011). A blue print for blue carbon: Toward an improved understanding of the role of vegetated coastal habitats in sequestering CO_2_. Front. Environ. Ecol..

[22] Wylie L., Sutton-Grier A.E., Moore A. (2016). Keys to successful blue carbon projects: Lessons learned from global case studies. Mar. Policy.

[23] White M., Bashmachnikov I., Aristegui J., Martins A. (2007). Physical processes and seamount productivity. Fish Aquat. Res. Ser..

[24] Barnes D.K.A., Sands C.J. (2017). Functional group diversity is key to Southern Ocean benthic carbon pathways. PLoS ONE.

[25] Barnes D.K.A., Sands C.J., Richardson A., Smith N. (2019). Extremes in benthic ecosystem services; Blue Carbon natural capital shallower than 1000 m in isolated, small and young Ascension island’s EEZ. Front. Mar. Sci..

[26] Souster T., Barnes D.K.A., Hopkins J. (2020). Variation in zoobenthic blue carbon in the Arctic’s Barents Sea shelf sediments. Phil. Trans. R. Soc. A.

[27] Schneider C.A., Rasband W.S., Eliceiri K.W. (2012). NIH Image to ImageJ: 25 years of image analysis. Nat. Methods.

[28] Langenkämper D., Zurowietz M., Schoening T., Nattkemper T.W. (2017). BIIGLE 2.0—Browsing and annotating large marine image collections. Front. Mar. Sci..

[29] Caselle J.E., Hamilton S.L., Davis K., Thompson C.D., Turchik A., Jenkinson R., Simpson D., Sala E. (2018). First quantification of subtidal community structure at Tristan da Cunha Islands in the remote South Atlantic: From kelp forests to the deep sea. PLoS ONE.

[30] North W.J., North W.J., Lehre J. (1971). Growth of individual fronds of the mature giant kelp *Macrocystis*. The Biology of Giant Kelp Beds (Macrocystis) in California.

[31] Diekmann G.S. (1978). Aspects of Growth and Production of *Laminaria pallida* off the Cape Peninsula. Master’s Thesis.

[32] Wickham S.B., Darimont C.T., Reynolds J.D., Staromski B.M. (2019). Species-specific wet-dry mass calibrations for dominant Northeastern Pacific Ocean macroalgae and seagrass. Aquat. Bot..

[33] Mora-Soto A., Palacios M., Macaya E.C., Gómez I., Huovinen P., Pérez-Matus A., Young M., Golding N., Toro M., Yaqub M. (2020). A high-resolution global map of giant Kelp (*Macrocystis pyrifera*) forests and intertidal Green Algae (*Ulvophyceae*) with sentinel-2 imagery. Remote Sens..

[34] Krumhansl K., Scheibling R. (2012). Production and fate of kelp detritus. Mar. Ecol. Prog. Ser..

[35] Krause-Jensen D., Lavery P., Serrano O., Marbà N., Masque P., Duarte C.M. (2018). Sequestration of macroalgal carbon: The elephant in the Blue Carbon room. Biol. Lett..

[36] Hill R., Bellgrove A., Macreadie P.I., Petrou K., Beardall J., Steven A., Ralph P.J. (2015). Can macroalgae contribute to blue carbon? An Australian perspective. Limnol. Oceanogr..

[37] De Clippele L.H., Rovelli L., Ramiro-Sánchez B., Kazanidis G., Vad J., Turner S., Glud R.N., Roberts J.M. (2021). Mapping cold-water coral biomass: An approach to derive ecosystem functions. Coral Reefs.

[38] Carbon Pricing Leadership Coalition (2019). Report of the High-Level Commission on Carbon Pricing and Competitiveness.

[39] Vecsei A. (2004). A new estimate of global reefal carbonate production including the fore-reefs. Glob. Planet. Chang..

[40] Roberts C.M. (2002). Deep impact: The rising toll of fishing in the deep sea. Trends Ecol. Evol..

[41] Santillo D., Johnston P.A. (2005). Marine protected areas (MPAs) as management tools to conserve seamount ecosystems. Proceedings of the International Conference on Governance and Management of Deep-Sea Fisheries.

[42] Howell K.L., Davies J.S., Narayanaswamy B.E. (2010). Identifying deep-sea megafaunal epibenthic assemblages for use in habitat mapping and marine protected area network design. J. Mar. Biol. Assoc. UK.

[43] Clark M.R., Bowden D.A. (2015). Seamount biodiversity: High variability both within and between seamounts in the Ross Sea region of Antarctica. Hydrobiology.

[44] Ross R.E., Howell K.L. (2013). Use of predictive habitat modelling to assess the distribution and extent of the current protection of ‘listed’ deep-sea habitats. Divers. Distrib..

[45] Ross L.K., Ross R.E., Stewart H.A., Howell K.L. (2015). The influence of data resolution on predicted distribution and estimates of extent of current protection of three ‘listed’ deep-sea habitats. PLoS ONE.

[46] Smale D.A., Moore P.J., Queirós A.M., Higgs N.D., Burrows M.T. (2018). Appreciating interconnectivity between habitats is key to blue carbon management. Front. Ecol. Environ..

[47] Lundsten L., Barry J., Cailliet G., Clague D., DeVogelaere A., Geller J. (2019). Benthic invertebrate communities on three seamounts off southern and central California, USA. Mar. Ecol. Prog. Ser..

[48] Levin L.A., Thomas C.L. (1989). The influence of hydrodynamic regime on infaunal assemblages inhabiting carbonate sediments on central Pacific seamounts. Deep Sea Res..

[49] O’Hara T.D., Tittensor D.P. (2010). Environmental drivers of ophiuroid species richness on seamounts. Mar. Ecol..

[50] Morgan N.B., Goode S., Roark E.B., Baco A.R. (2019). Fine scale assemblage structure of benthic invertebrate Megafauna on the north pacific seamount Mokumanamana. Front. Mar. Sci..

[51] Queirós A.M., Hiddink J.G., Kaiser M.J., Hinz H. (2006). Effects of chronic bottom trawling disturbance on benthic biomass, production and size spectra in different habitats. J. Exp. Mar. Biol. Ecol..

[52] Watson R., Kitchingman A., Cheung W., Pitcher T.J., Morato T., Hart P.J.B., Clark M.R., Haggan N., Santos R.S. (2007). Catches from world seamount fisheries. Seamounts: Ecology, Fisheries & Conservation.

[53] Clark M.R., Rowden A.A., Schlacher T., Williams A., Consalvey M., Stocks K.I., Rogers A.D., O’Hara T.D., White M., Shank T.M. (2010). The ecology of seamounts: Structure, function, and human impacts. Ann. Rev. Mar. Sci..

[54] Van Oevelen D., Duineveld G., Lavaleye M., Mienis F., Soetaert K., Heip C.H.R. (2009). The cold-water coral community as a hot spot for carbon cycling on continental margins: A food-web analysis from Rockall Bank (northeast Atlantic). Liminol. Oceanogr..

[55] Sabatier P., Reyss J.L., Hall-Spencer J.M., Colin C., Frank N. (2012). 210Pb-226Ra chronology reveals rapid growth rate of *Madrepora oculata* and *Lophelia pertusa* on world’s largest cold-water coral reef. Biogeosciences.

[56] Mallela J. (2013). Calcification by reef-building sclerobionts. PLoS ONE.

[57] Lunden J.J., McNicholl C.G., Sears C.R., Morrison C.L., Cordes E.E. (2014). Acute survivorship of the deep-sea coral Lophelia pertusa from the Gulf of Mexico under acidification, warming, and deoxygenation. Front. Mar. Sci..

[58] Koslow J., Gowlett-Holmes K., Lowry J., O’Hara T., Poore G., Williams A. (2001). Seamount benthic macrofauna off southern Tasmania: Community structure and impacts of trawling. Mar. Ecol. Prog. Ser..

[59] Nolan E., Barnes D., Brown J., Downes K., Enderlein P., Gowland E., Brickle P. (2017). Biological and physical characterization of the seabed surrounding Ascension Island from 100–1000 m. J. Mar. Biol. Ass. UK.

